# Range extension for the common dolphin (*Delphinus* sp.) to the Colombian Caribbean, with taxonomic implications from genetic barcoding and phylogenetic analyses

**DOI:** 10.1371/journal.pone.0171000

**Published:** 2017-02-13

**Authors:** Nohelia Farías-Curtidor, Dalia C. Barragán-Barrera, Paula Alejandra Chávez-Carreño, Cristina Jiménez-Pinedo, Daniel M. Palacios, Dalila Caicedo, Fernando Trujillo, Susana Caballero

**Affiliations:** 1 Conjunto Caeli Reservado, Cajicá, Colombia; 2 Laboratorio de Ecología Molecular de Vertebrados Acuáticos LEMVA, Departamento de Ciencias Biológicas, Universidad de los Andes, Laboratorio J-202, Bogotá, Colombia; 3 Fundación Macuáticos Colombia, Medellín, Colombia; 4 Nicholas School of the Environment, Duke University, Durham, North Carolina, United States of America; 5 Zona Rural - Piedras Coloradas S/N. Las Grutas, Río Negro, Argentina; 6 Marine Mammal Institute, Oregon State University, Hatfield Marine Science Center, Newport, Oregon, United States of America; 7 Fundación Omacha, Bogotá, Colombia; Academia Sinica, TAIWAN

## Abstract

The nearest known population of common dolphins (*Delphinus* sp.) to the Colombian Caribbean occurs in a fairly restricted range in eastern Venezuela. These dolphins have not been previously reported in the Colombian Caribbean, likely because of a lack of study of the local cetacean fauna. We collected cetacean observations in waters of the Guajira Department, northern Colombia (~11°N, 73°W) during two separate efforts: (a) a seismic vessel survey (December 2009—March 2010), and (b) three coastal surveys from small boats (May—July 2012, May 2013, and May 2014). Here we document ten sightings of common dolphins collected during these surveys, which extend the known range of the species by ~1000 km into the southwestern Caribbean. We also collected nine skin biopsies in 2013 and 2014. In order to determine the taxonomic identity of the specimens, we conducted genetic barcoding and phylogenetic analyses using two mitochondrial markers, the Control Region (mtDNA) and Cytochrome b (Cytb). Results indicate that these specimens are genetically closer to the short-beaked common dolphin (*Delphinus delphis*) even though morphologically they resemble a long-beaked form (*Delphinus* sp.). However, the specific taxonomic status of common dolphins in the Caribbean and in the Western Atlantic remains unresolved. It is also unclear whether the distribution of the species between northern Colombia and eastern Venezuela is continuous or disjoined, or whether they can be considered part of the same stock.

## Introduction

Common dolphins (genus *Delphinus*) are widely distributed in all tropical and temperate oceans around the world. However, details of their distribution are not well described because until 1994 all common dolphins were regarded as single species, *D*. *delphis*, despite knowledge of the existence of long-beaked and short-beaked morphotypes [1, 2]. Morphological features that include body coloration, teeth number, vertebral number and the rostral length: zygomatic width ratio, and genetic comparisons between long-beaked and short beaked morphotypes using mitochondrial markers in the Eastern North Pacific established the long-beaked morphotype as *D*. *capensis*, a separate species from the short-beaked morphotype, *D*. *delphis* [1, 3]. Nevertheless, the separation of the genus *Delphinus* into a short-beaked and a long-beaked species on a global scale is not clear under these morphological, phylogenetic, and genetic evidence, and therefore taxonomy of this species remains controversial [4, 5, 6, 7, 8].

Rostral length measures from California common dolphin populations have been used as a reference to identify these two species in the rest of the world. However, not all common dolphins from different parts of the world fit in the reference measures [6, 9, 10, 11]. Furthermore, several phylogenetic studies have found that the two species are not reciprocally monophyletic when taking into account common dolphins from different ocean basin [4, 5, 8, 12, 13, 14], making *Delphinus* taxonomy unclear at the worldwide level. Additionally, in what respects to population genetics studies, it is known that the there is significant genetic differentiation between populations present in different oceans basins and in different regions of the Atlantic Ocean [4, 8, 15]. Yet, genetic structure among populations has not been detected on each side of the Atlantic Ocean [4, 8, 15, 16].”

In the Western Atlantic the identity and taxonomic status of the genus is still unclear because early observations mistakenly identified as common dolphins the similarly colored Clymene (*Stenella clymene*) and spinner (*S*. *longirostris*) dolphins [17]. Specifically, within the Caribbean Basin, erroneous reports of *Delphinus* have been common in areas like Cuba, where Cuní [18] and Aguayo [19] described this genus from stranded animals that were most likely Clymene or spinner dolphins [20]. Also, although both long and short-beaked morphotypes occur in the Western Atlantic [17], the corresponding taxonomic assignment (*D*. *capensis* and *D*. *delphis*) only applies to the Eastern North Pacific populations since the results of Heyning & Perrin [1] have not been validated for common dolphin populations elsewhere [4, 7, 8]. Particularly in the Western South Atlantic, the long-beaked morphotype has been described only based on a report published by Casinos [21], who conducted craniometric measurements on ten specimens from Venezuela, Brazil and Argentina, of which only six corresponded to the long-beaked morphotype [1, 7]. In fact, initial morphological and genetic studies of Western South Atlantic specimens suggest that a different taxonomic classification should be considered in this region [4, 11, 14, 22, 23]. For this reason, and because the global taxonomy of long-beaked common dolphins remains unresolved, we refer to the animals observed in this report as *Delphinus* sp.

Only one population of *Delphinus* has been confirmed for the Caribbean Basin, the “Venezuelan Stock” [11, 22]. This is an apparently isolated, coastal population of common dolphins occurring mainly in the Cariaco Basin of eastern coast of Venezuela [17]. The species is common in waters around Margarita Island, Mochima National Park, the Gulf of Cariaco, and the Araya and Paria peninsulas [23]. Isolated records from the Gulf of Venezuela to the west and from Trinidad to the east have been considered as vagrants by Jefferson [17]. Here we report on ten sightings of common dolphins from coastal waters of northern Colombia that represent a new species record in the country and that also extend the known distribution range of the species from the southeastern to the southwestern Caribbean. In addition, the main purpose of this study was to use genetic barcoding and phylogenetic analyses to provide a taxonomic identification of the common dolphins from Colombia, using mitochondrial DNA markers such as Cytochrome *b* (Cyt*b*) and Control Region (D-loop)

## Methods

### Visual surveys and biopsy sampling

Systematic marine wildlife observation aimed at collecting baseline information was recently initiated in Colombia in response to proposed economic development initiatives like offshore oil and gas drilling and industrial port building. As part of these monitoring programs, Fundación Omacha has placed observers on two separate sampling platforms off northern Colombia. The first set of observations was collected aboard a 54-m seismic vessel from December 2009 to March 2010 (93 days). The routes were systematic with transects defined within oil exploration blocks (12°00’W to 11°20’W and 72°60’N to 73°60’N and 14 to 60 miles from the coast). Marine wildlife observation was conducted during 12 hours of daylight from the bridge of the vessel, 5 m above the sea surface using the naked eye as well as 7x50 mm binoculars.

The second set of observations was collected during small-scale coastal surveys conducted out of Mingueo, municipality of Dibulla, Guajira Department (11°00’N, 73°40’W), between May and July 2012 (65 days), May 2013 (seven days) and May 2014 (seven days). Two boats were used for this purpose, a 6-m wooden boat with a 40-hp outboard engine and a 9-m fiberglass boat with two 100-hp outboard engines. Surveying was conducted during morning (0700–1200 h) and afternoon (1400–1730 h) trips, using the naked eye and 7x50 mm binoculars. These trips departed from the mouth of the Cañas River and covered the stretch of coast between the Ancho River to the west and the Port of Brisa to the east. The routes followed were not systematic and depended on favorable weather conditions. Daily trips searched 10 to 12 miles into open sea in different directions. During the 2013 and 2014 surveys skin samples were obtained from wild dolphins using a remote biopsy system consisting of small darts fired from a modified rifle (PAXARMS) [24]. This system allows penetration of dolphin epidermis leaving behind a small wound [25]. However, the effect on dolphins is low, because the polycarbonate body of dart to spread the impact over a wider area and therefore, reduce the risk of injury by penetration [24, 25]. This methodology was approved by the Universidad de los Andes CICUAL-Comité Institucional para el cuidado y uso de animales de laboratorio- (Institutional laboratory animal care and use committee). Samples were preserved in 70% alcohol and later stored at -20°C [26] for laboratory analysis. These samples were collected under Resolution 1177 Permit (Permiso Marco) for Specimen Collection of Wildlife Biodiversity Non Commercial Purposes of Scientific Research. This permit was provided by the National Authority for Environmental Licenses (ANLA) to Universidad de los Andes.

### DNA extraction, PCR amplification, sequencing, and molecular sexing

We used the DNeasy kit (QIAGEN, Valencia, CA, USA) to extract DNA from nine skin samples of common dolphins obtained in La Guajira during this study. Primers t-Pro-whale M13Dlp1.5 (5′-TGTAAAACGACAGCCAGTTCACCCAAAGCTGRARTTCTA-3′) and Dlp8G (5′- GGAGTACTATG TCCTGTAACCA-3′) were used to amplify a portion of 650 basepairs (bps) of Control Region (D-loop), through the polymerase chain reaction (PCR), following amplification conditions proposed by Baker *et al*. [27]. We also amplified a portion of 400 bps of the Cytochrome *b* (Cyt*b*) gene in both forward and reverse directions, using the primers Tglu (5′-TGACTTGAARAACCAYCGTTG-3′) and CB2 (5′-CCCTCAGAATGATATTTGTCCTCA-3′), and following the same PCR conditions used to amplify the D-loop region.

According to Amaral *et al*. [5], to identify related species whose process of lineage sorting is not complete, the Cyt*b* gene is more reliable for taxonomic identification than the Control Region, and because delphinids have complex phylogenetic relationships [12, 28], we included both markers to confirm the species identification. Successfully amplified PCR products were purified following a Polietilenglicol protocol (PEG 20%), and DNA sequencing was conducted using the Sanger sequencing method [29]. All samples were sexed following the protocol by Gilson et al. [30].

### Genetic barcoding

Sequences obtained from the D-loop and Cyt*b* were edited and aligned manually using the software Geneious v4.8.5. [31]. In order to identify the species, we conducted a barcoding and cluster analysis advance with 1000 replicates on the “DNA Surveillance”, a validated and curated database of cetacean sequences at the University of Auckland [32, 33]. This analysis provides a species identification tree indicating what species is most closely related to the problem sequence. The software uses phylogenetic algorithms, providing evolutionary distances between problem (query) and reference sequences [32]. To compare data shown by “DNA Surveillance”, we conducted a nucleotide blast (blastn) on the National Center for Biotechnology Information (NCBI) website [34] using the megablast program in order to find highly similar sequences for our query. For NCBI results we took into account the affinity between the match of sequences and the Expected value (E), which describes the random background noise, being lower or closer to zero if the match between sequences is significant [34].

### Phylogenetic analyses

A total of 347 bp consensus sequence for Cytb was compiled, analyzed and compared with 190 published sequences in GenBank from around the world (Table 1). As well, a 447 bp consensus sequence for D-loop was compiled, analyzed and compared with 190 published sequences from various locations worldwide (Table 1). Haplotypes were defined using the R script RemoveRedundantTaxa [35], which is a substitute for a MacClade utility.

**Table 1:** Published sequences of common dolphins used in this study for Cyt*b* and Control Region (D-loop).

mtDNA marker	Location	References	GenBank Assesion numbers
Cyt*b*			
	Eastern North Atlantic	[5, 36, 37]	DQ378138- DQ378164;
			JX264574- JX264582;
			KC297722-KC297725;
			KC297765-KC297766;
			EU517699- EU517700
	Western North Atlantic	[36,37]	KC297742- KC297743;
			EU517701;EU517707
	Eastern Central Atlantic	[36]	KC297759-KC297764
	Eastern South Atlantic	[36]	KC297744-KC297758
	Eastern North Pacific	[3, 12, 36]	DDU02665-DDU02676;
			AF084084-AF084088;
			KC297710-KC297721
	Western South Pacific	[36]	JX264641-JX264702;
			KC297726-KC297741
	Eastern South Pacific	[37, 38]	HM572297-HM572302;
			EU517697
D-loop			
	Eastern North Atlantic	[5, 16]	DQ378096-DQ378137;
			EF682507-EF682649
	Eastern North Pacific	[3, 39]	DDU01956-DDU02676;
			HE680096-HE680202
	Australia	[40, 41]	FJ175416-FJ175450;
			HQ223451-HQ223479

jModelTest v2.1.7 [42, 43] was used to find the best-fitting model of nucleotide substitution among the sequences of both mitochondrial markers haplotypes. In the case of Cyt*b* the best model was HKY+I, and for the control region the best model was K80+G. Each gene was analyzed independently since there were no sequences for both genes from the same individuals in all locations. Two different trees were obtained: one for Cyt*b* and one for D-loop. The software package BEAST [44] was used to generate the phylogenetic relationship tree, establishing *Globicephala macrorhynchus* as outgroup. The program was ran with 10 million MCMC generations sampling every 1,000 generations, Yule process was specified as the species tree prior, and a Normal distribution for the tmrca. The HKY+I and K80+G models were used for Cyt*b* and D-loop respectively. An uncorrelated lognormal distribution relaxed molecular clock was chosen. The program TRACER v1.6 [45] was used to check mixing and convergence of parameters and posterior distribution against generations. Finally, TreeAnnotator v1.8.2 [46] was run to summarize the trees obtained in one consensus tree.

## Results

### Visual and photographic documentation

**Fig 1:**
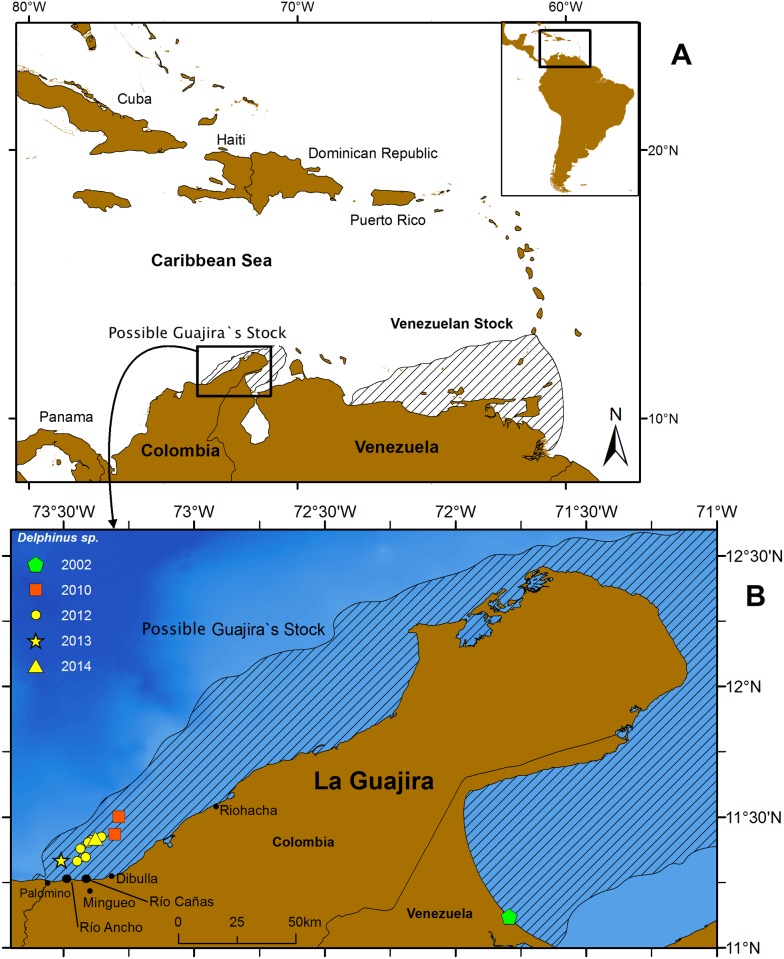
Location of the first records of common dolphins (*Delphinus* sp.) in the Colombian Caribbean. Map A shows the potential distribution area of possible Guajira´s stock and the range of the Venezuelan Stock. Map B shows sightings of common dolphins in the Guajira Peninsula. Green symbols indicate strandings, red symbols indicate sightings collected from a seismic vessel, and the yellow symbols indicate sightings collected during small-boat coastal surveys.

Here we report a total of ten sightings of common dolphins (*Delphinus* sp.) (Table 2). All sightings were obtained in coastal waters of the Guajira Department, no more than 12 miles from shore. Two sightings were made from the seismic vessel in one of the oil exploration blocks off the lower Guajira Peninsula, on 19 and 25 February 2010, respectively (Fig 1). Other species seen while surveying this block included pantropical spotted dolphins (*Stenella attenuata*), spinner dolphins (*S*. *longirostris*), striped dolphins (*S*. *coeruleoalba*), Atlantic spotted dolphins (*S*. *frontalis*) and common bottlenose dolphins (*Tursiops truncatus*). Eight additional sightings of common dolphins were made during the small-scale coastal surveys off the Dibulla municipality between May and June 2012, in May 2013, and in May 2014 (Fig 1). Other species seen during these surveys included Atlantic spotted dolphins, spinner dolphins, and common bottlenose dolphins.

**Table 2:** Common dolphins sighting data (date, location and water depth).

Date (D/M/Y)	Location	Depth (m)
19/02/2010	73° 18' 6'' W 11° 26' 1'' N	32
25/02/2010	73° 17' 9'' W 11° 30' 5'' N	70
17/05/2012	73° 23' 37'' W 11° 24' 36'' N	100
09/06/2012	73° 26' 48'' W11° 19' 51'' N	46
12/06/2012	73° 21' 13'' W 11° 25' 26'' N	43
16/06/2012	73° 24' 51'' W 11° 20' 46'' N	40
18/06/2012	73° 24' 15'' W 11° 24' 8'' N	81
30/06/2012	73° 26' 1'' W 11° 22' 43'' N	58
24/05/2013	11° 19' 70.8'' W 73° 29' 85.2‴N	Not available
17/05/2014	11° 25' 00.6'' W 73° 22' 33'' N	Not available

**Fig 2:**
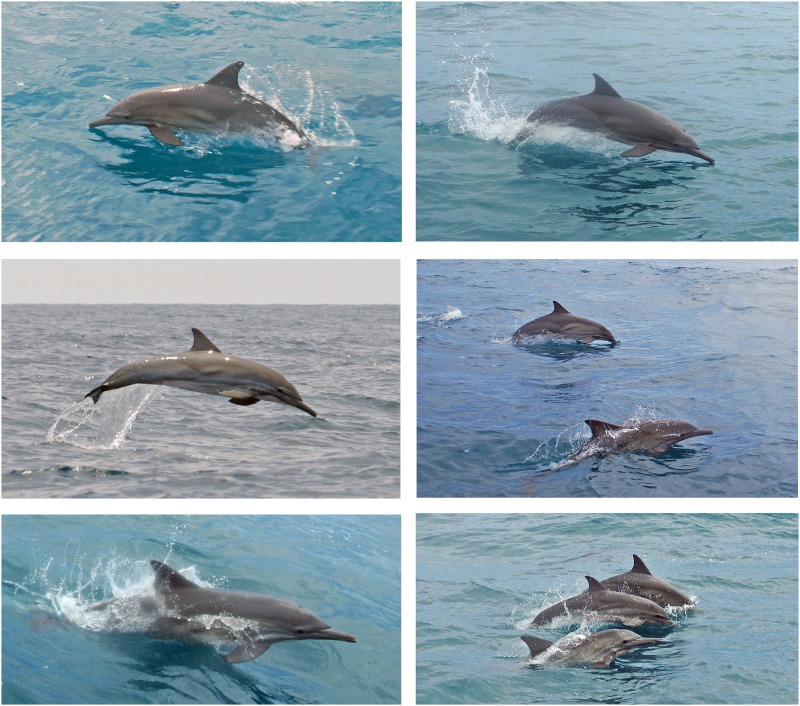
Photographs of common dolphins (*Delphinus* sp.) taken in the northern Colombia. These photos were taken the 9^th^ and 17^th^ May 2012 off the lower Guajira Peninsula near Mingueo, municipality of Dibulla. The diagnostic external morphology and coloration pattern of the genus are evident in all images. In addition, although the pattern is rather muted, note how the tan-coloured thoracic patch is interrupted by a secondary dark stripe that runs forward and upward from the eye-to-anus stripe.

Exact group size counts were not made, but the observers reported an overall group size ranging between 20 and 60 animals in all sightings, including adults, juveniles and calves. All animals in these sightings were identified as common dolphins based on good photographs (Fig 2). Diagnostic features included a long rostrum, a somewhat flattened melon, a dark cape crisscrossing light patches on the thorax forming an “hourglass”, and a complex system of stripes originating in the chin and gape and running toward the eye, flipper and anus [17, 47]. These dolphins had a duller and more indistinct coloration than is typical of the short-beaked common dolphin (*D*. *delphis*), which according to Jefferson *et al*. [17] does not occur in the Caribbean Sea.

### Barcoding analyses

**Fig 3:**
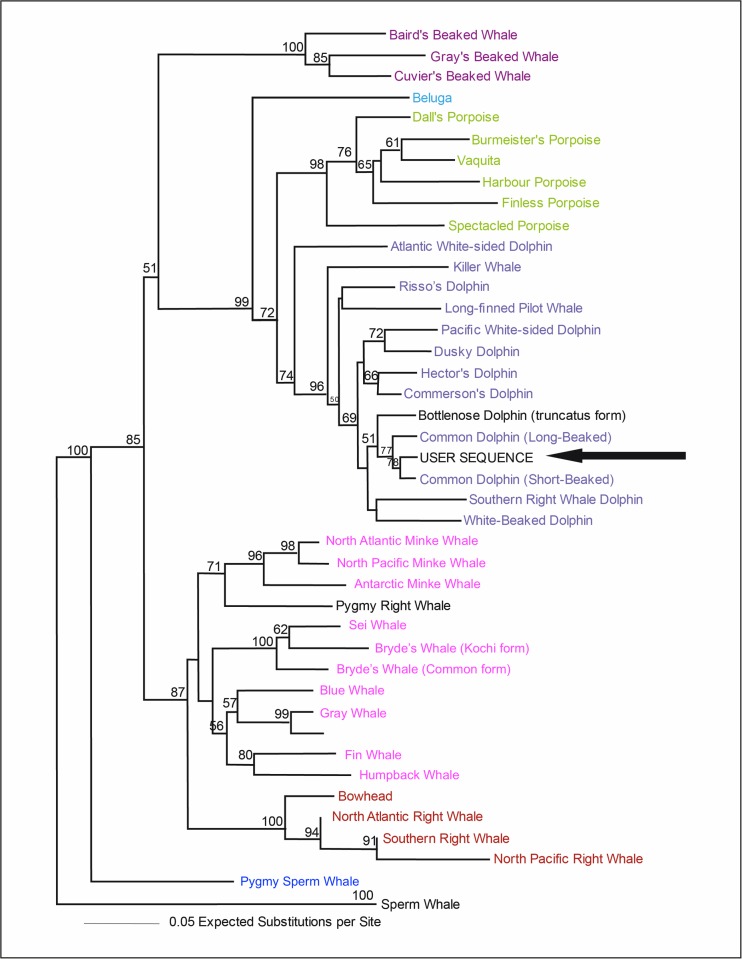
Tree based on the Surveillance barcoding results with the D-loop gene. Sequences obtained from samples from La Guajira (user sequence) showed affinity with the reference sequences. Evolutionary distance with D-loop sequences ranged between 0.0179 and 0.023 changes/site for short-beaked common dolphins and between 0.0333 and 0.0385 changes/site for long-beaked common dolphins. Values below the branches correspond to neighbor-joining (NJ) bootstrap support values [32].

All nine samples were successfully sexed and five females and four males were identified. Barcoding analyses on “DNA Surveillance” showed that the common dolphin sequences from this study were more closely related to *D*. *delphis* than to *D*. *capensis*. Evolutionary distance with D-loop sequences ranged between 0.0179 and 0.023 changes per site for short-beaked common dolphins and between 0.0333 and 0.0385 changes per site for long-beaked common dolphins (Fig 3). Significant Blastn results with NCBI (E = 0) also showed that all sequences were more closely related to *D*. *delphis* (99% affinity between sequences for all problem sequences) than to *D*. *capensis* (97% affinity between sequences for all problem sequences).

**Fig 4:**
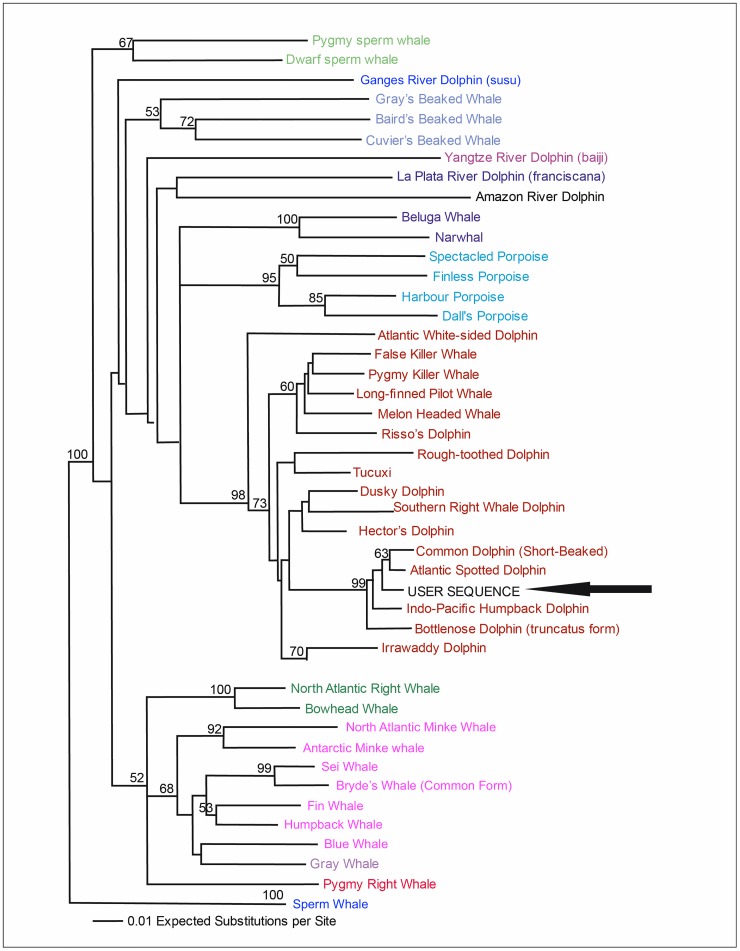
DNA Surveillance barcoding results with the Cytb gene. Sequences obtained from samples from La Guajira (user sequence) showed affinity with the reference sequences. Evolutionary distance with Cytb sequences ranged between 0.0197 and 0.0228 changes/site for short-beaked common dolphins (*Delphinus delphis*). Values below the branches correspond to neighbor-joining (NJ) bootstrap support values [32].

In contrast, barcoding analyses on “DNA Surveillance” using Cyt*b* sequences showed that the problem sequences were related to the short-beaked common dolphin (evolutionary distances ranging between 0.0197 and 0.0228 changes per site) and to the Atlantic spotted dolphin (with evolutionary distances ranging from 0.0123 changes/site to 0.0142 changes/site) (Fig 4). Blastn results with NCBI, although significant (E = 0), were inconclusive since all sequences were related to *Stenella* spp., *Tusiops* spp. and *Delphinus* spp. specimens. Due to high intraspecific diversity and low interspecific divergence within the Delphininae subfamily [28], these results are not surprising since genus *Delphinus* is part of “the STD complex” (*Stenella*, *Tursiops* and *Delphinus*), and consequently it is difficult to resolve the relationship among these species using only Cyt*b* sequences [48].

### Phylogenetic analyses

**Fig 5:**
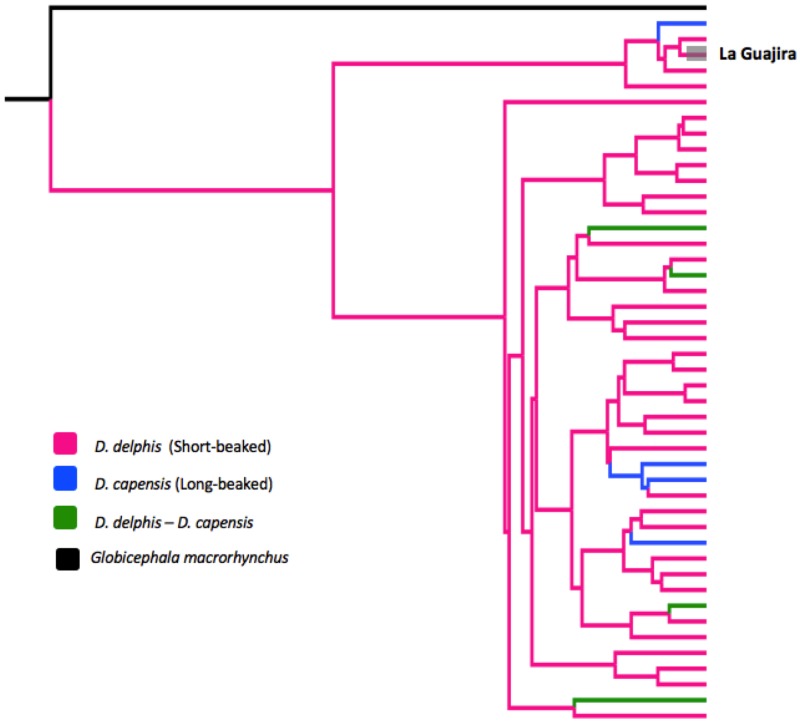
Phylogenetic reconstruction of common dolphins Cytochrome *b* (Cyt*b*) worldwide haplotypes. Cyt*b* haplotype identified insamples from La Guajira is highlighted in grey.

For Cyt*b* a total of eight sequences were amplified from La Guajira samples. Sequences of 347 bp were analyzed and compared with 190 *Delphinus* sp. published sequences, defining 45 haplotypes. A rooted *G*. *macrorhynchus* phylogenetic tree was reconstructed using these 45 haplotypes (Fig 5). Sequences from La Guajira conformed one haplotype that was previously reported for *D*. *delphis* from the Eastern North Atlantic, and this haplotype was grouped with a *D*. *delphis* clade. Long-beaked common dolphin haplotypes represent a polyphyletic group in this phylogeny, since they are nested with different clades all over the tree. Moreover, there are four haplotypes shared between sequences of *D*. *delphis* and *D*. *capensis*, which are as well nested with different clades all over the tree. There was no geographical structuring in the phylogeny, so haplotype location was not specified in Fig 5.

**Fig 6:**
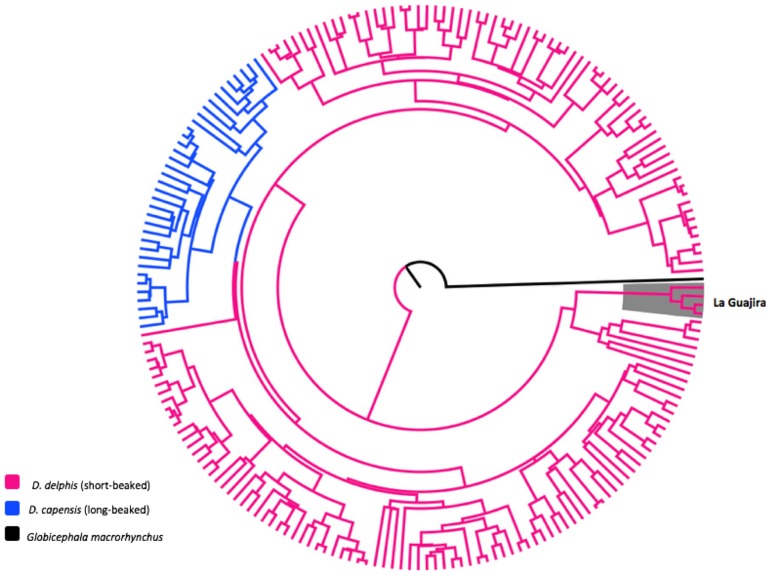
Phylogenetic reconstruction of common dolphins Control Region (D-loop) worldwide haplotypes. Control Region (D-loop) haplotypes identified in samples from La Guajira are highlighted in grey.

For the control region (D-loop), a total of nine sequences were obtained from La Guajira. Among the 362 sequences analyzed, including amplified sequences and published sequences, 202 haplotypes were identified. A phylogenetic relationship tree was constructed using these haplotypes as shown in Fig 6. Sequences from La Guajira contained four unique haplotypes not reported before. All of these unique haplotypes were nested within the *D*. *delphis* complex in the phylogeny. In this phylogenetic reconstruction, *D*. *capensis* haplotypes represent a monophyletic clade; however, the long-beaked common dolphin clade is also nested within the short-beaked common dolphin complex. No geographical structure was present in the phylogeny, thus it was not taken into account in Fig 6.

## Discussion

Recent survey work in a previously unexplored area of the Colombian Caribbean in 2010, 2012, 2013, and 2014 has yielded ten sightings of the common dolphin, a new species record for the country. These sightings also extend the known distribution range of the species to the southwestern Caribbean and provide new information on their local distribution and external appearance. The global distribution of common dolphins largely coincides with temperate and tropical coastal areas influenced by wind-driven upwelling [17]. In this respect the occurrence of common dolphins off the Guajira Peninsula is not unexpected, as the area is known for persistent upwelling and high productivity [49, 50, 51, 52, 53, 54].

The nearest population of common dolphins inhabits the Cariaco Basin off the central/eastern coast of northern Venezuela [17, 23], some 700–1400 km to the east of the area surveyed in this study. A stranding record from western Venezuela near the Colombian border [55] was considered unusual in the review of Jefferson [17]. However, in light of the new records presented here it is possible that the species has a continuous distribution from eastern Venezuela to northern Colombia, especially considering that the entire northern coast of South America along the Caribbean is subject to upwelling [48, 51, 56]. Alternatively, two populations may occur in the southern Caribbean, one in the east and one in the west (as drawn in Fig 1A), associated with the areas of most intense upwelling and highest productivity [57]. These alternative scenarios remain to be elucidated through further survey work on a regional scale.

Regarding the taxonomic identity of the animals in this study, although their external appearance would suggest a long-beaked form, genetic barcoding and phylogenetic analyses indicated that they are more closely related to the short-beaked form. In fact, *Delphinus* from the Western South Atlantic do not show a complete affinity to Heyning & Perrin’s [1] rostrum length zygomatic width ratio proposition to distinguish the two species [14], since there are intermediate measures in specimens from this zone [11, 22]. Consequently, our results support the idea that the *capensis* epithet only applies to the species occurring in the Eastern North Pacific, and that the taxonomic identity of other long-beaked forms from around the world, including the Western South Atlantic, requires revision as has also been determined by multiple previous studies [4, 11, 14, 22].

Genetic differences reported by Rosel *et al*. [3] between the long-beaked morphotype and the short-beaked form included fixed nucleotide substitutions present only in sequences of mitochondrial DNA (D-loop and Cyt*b*) of *D*. *capensis*. However, our sequences do not share these fixed sites but share similarities with *D*. *delphis* sequences, and were grouped with the short-beaked morphotype. Moreover, we found that the two species *D*. *delphis* and *D*. *capensis* do not show reciprocal monophyly. For the Cyt*b* tree, *D*. *capensis* haplotypes clearly represent a polyphyletic group, and more interesting, four haplotypes were shared between *D*. *capensis* and *D*. *delphis* sequences, indicating no real differentiation between both species for the Cyt*b*. For the D-loop phylogenetic tree, no shared haplotypes between both species were found and *D*. *capensis* represented a monophyletic clade. Nevertheless, the *D*. *capensis* clade was nested within the *D*. *delphis* complex, indicating no taxonomic differentiation between the two species.

Similarly, many phylogenetic studies using mitochondrial markers have shown *D*. *capensis* sequences nested with *D*. *delphis* sequences, demonstrating that there is no reciprocal monophyly between the two common dolphin morphotypes [4, 5, 8, 12, 14, 36]. For example, Cunha *et al*. [14] found that common dolphins in the Western South Atlantic form a single species. In addition, phylogenetic results of Natoli *et al*. [4] using nuclear markers (microsatellites) showed no differentiation among populations inhabiting the same area of an ocean basin. For instance, dolphins from the Western North Atlantic do not show genetic differences with individuals from Argentina, but show some differences with dolphins from the Eastern North Atlantic. However, Amaral *et al*. [5] reported one unique haplotype shared between one dolphin from Argentina with *D*. *delphis* individuals from the Eastern North Atlantic populations, suggesting migration of the short-beaked morphotype to the Western South Atlantic. In summary, phylogenetic results support the assumption that the long-beaked morphotype could be the result of positive selection related to an adaptation to coastal environments, and for this reason the long-beaked morphotype is nested in the same clade but with *D*. *delphis* sequences.

It has been suggested that convergent evolution has led to the existence of different long-beaked morphotypes in different locations in the world [4, 5, 36], but this leaves no clear conclusion about whether both species can be truly recognized in all the ocean basins, or just in the Eastern North Pacific. A study in Mauritania showed skull differentiation in common dolphins to be related to niche segregation rather than to speciation [6], suggesting as well to have caution when considering long and short-beaked common dolphins from outside the Eastern North Pacific. Consequently, positive selection of the long-beaked morphotype could be explained by niche segregation due to habitat use and prey capture by the dolphins [6]. Because the long-beaked common dolphin is distributed in coastal areas and the short-beaked common dolphin occupies both inshore and offshore areas [1], diet should be different between both morphotypes, and consequently, the long-beaked form could originate independently in different regions [4], perhaps due to feeding ecology [6, 9, 58]. However, despite the distribution of *D*. *capensis* ranging in coastal areas [1, 17], some studies have found that dolphins with longer beaks not only can occur in deep waters, but that they also feed on prey associated with offshore areas [6, 59]. Also, in some coastal areas off Uruguay and Mauritania, where the long-baked morphotype is reported, some studies have found both forms distributed together not only inshore but also in offshore areas [6, 7]. In addition, reports of common dolphins in southern Brazil and Argentina have been made in offshore areas with depths ranging between 71 to 1435 m, and according to morphological analyses, both morphotypes are distributed in these areas [22]. Consequently, it is possible that both morphotypes are distributed together in many areas worldwide or at least in the Western South Atlantic. Therefore the Eastern North Pacific maybe a unique region where the two morphotypes occupy different areas in coastal and offshore waters [1, 3, 58].

The main assumption that supports the idea that *D*. *capensis* is distributed in the Western South Atlantic is based on morphological analyses of cranial measurements: six skulls from animals found in Argentina, Brazil and Venezuela correspond to the long-beaked morphotype according to Casinos [21], and three skulls of specimens found in Brazil and described as the nominal species *Delphinus microps* [60], were recognized as *D*. *capensis* by Heyning & Perrin [1]. Nevertheless, the small sample size of common dolphins skulls in the Western South Atlantic does not allow testing of either hypothesis. However, a recent study with 59 skulls collected in Brazil, Argentina, and Uruguay showed that both morphotypes are distributed in the Western South Atlantic, and it even reported three skulls with intermediate rostral length-zygomatic width radio [22]. Similarly, another study based on the analysis of 29 skulls from the Western South Atlantic shows that although *D*. *capensis* is present in southern Brazil (Rio de Janeiro and Rio Grande do Sul) and Argentina, and *D*. *delphis* is also distributed in the latter country [14]. Consequently, it is difficult to separate both species (*D*. *capensis* and *D*. *delphis*) using only morphometric data. In fact, due to high intraspecific diversity and low interspecific divergence of Delphininae subfamily, some cranial characters are not shared by all of the species in the same genus from *Delphinus*, *Stenella* or *Tursiops*, because these genus conform “the STD complex”. Thus, it is necessary for other kinds of studies (e.g. genetic, ecological) to determine what morphotype of common dolphin is present in the Western South Atlantic. However, particularly for genetic studies, mitochondrial DNA is not sufficient to determine the relationship among specimens from the STD complex [48], and it is necessary to include other genetic markers to determine evolutionary relationships in these dolphins [48, 61].

Further morphological, ecological, and genetic studies of these animals should provide a greater understanding of their taxonomic identity and their relation to other populations in the Western South Atlantic. This taxonomic uncertainty has implications for the management and conservation of common dolphin populations, since the conservation status for *D*. *delphis* and *D*. *capensis* might change if they are considered a single or separate species throughout their range. In this respect, the International Whaling Commission has recommended strongly that research efforts focus on determining the taxonomic identity of the genus *Delphinus* in the Western South Atlantic [62]. Monitoring work off northern Colombia should be continued in order to obtain baseline information about the status of the populations and the potential effects of several mineral extraction activities being proposed for the area in order to inform appropriate management and conservation measures.
